# Signs and symptoms attributed to urinary tract infections in nursing home residents across eight European countries

**DOI:** 10.1007/s41999-026-01427-9

**Published:** 2026-02-13

**Authors:** Marie Theut, Jette Nygaard Jensen, Valeria Antsupova, Malene Plejdrup Hansen, Laura vallejo-Torres, Carl Llor, Ana Garcia-Sangenis, Ramon Monfà, Nina Sodja, András Bálint, Lina Jaruseviciene, Christos Lionis, Anna Kowalczyk, Helena Glasova, Jesper Lykkegaard

**Affiliations:** 1 Research Unit for Antibiotic Stewardship and Implementation, Department of Clinical Microbiology, Copenhagen University Hospital - Herlev and Gentofte, Herlev, Denmark; 2 Research Unit for General Practice, Department of Public Health, University of Southern Denmark, Odense, Denmark; 3 Department of Clinical Microbiology, Copenhagen University Hospital –Herlev and Gentofte, Herlev, Denmark; 4 Center for General Practice at Aalborg University, Gistrup, Denmark; 5 Department of Quantitative Methods in Economics and Management, Universidad de Las Palmas de Gran Canaria, Las Palmas de Gran Canaria, Spain; 6 Institut Català de La Salut, Barcelona, Spain; 7 CIBER de Enfermedades Infecciosas., Instituto de Salud Carlos III, Madrid, Spain; 8 Fundació Institut Universitari Per a La Recerca a L’Atenció Primària de Salut Jordi Gol, Barcelona, Spain; 9 Department of Family Medicine, Faculty of Medicine, University of Ljubljana, Ljubljana, Slovenia; 10 Szeged Autumn Nursing Home, Szeged, Hungary; 11 Department of Family Medicine, Lithuanian University of Health Sciences, Kaunas, Lithuania; 12 Department of Social Medicine, School of Medicine, University of Crete, Heraklion, Greece; 13 Centre for Family and Community Medicine, The Faculty of Health Sciences, The Medical University of Lodz, Lodz, Poland; 14 Department of Clinical Pharmacology, Faculty of Medicine, Slovak Medical University in Bratislava, Bratislava, Slovakia

**Keywords:** Urinary tract infections, Nursing home residents, Symptoms

## Abstract

**Purpose:**

This study examines which signs and symptoms lead to antibiotic treatment for suspected urinary tract infections in nursing home residents across eight European countries. Understanding these factors may clarify diagnostic practices and help reduce overdiagnosis.

**Methods:**

From February to April 2024, nursing home staff in Denmark, Greece, Hungary, Lithuania, Poland, Slovakia, Slovenia, and Spain completed a registration chart for each resident treated with antibiotics. The chart collected data on treatment indication, risk factors for urinary tract infection, and signs and symptoms attributed to the infection. Descriptive analysis was conducted to examine variation in signs and symptoms attributed to urinary tract infections across countries.

**Results:**

This study included 9718 residents across 109 nursing homes. During the study period, 975 antibiotic treatments were initiated on suspicion of urinary tract infection in residents without an indwelling urinary catheter. The median age of residents receiving treatment was 86 years, and 76% were women. Substantial variation was observed in the signs and symptoms attributed to urinary tract infections. In some countries, general symptoms, such as changes in behavior and confusion, predominated; in others, urinary tract symptoms were more common; while in others, changes in urine were most frequently reported.

**Conclusions:**

The marked variation in signs and symptoms attributed to urinary tract infections across countries highlights both the diagnostic complexity and the lack of consensus regarding diagnostic approaches in nursing home settings. This uncertainty may contribute to overdiagnosis and inappropriate antibiotic prescribing. Greater standardization and improved diagnostic guidance are warranted.

**Supplementary Information:**

The online version contains supplementary material available at 10.1007/s41999-026-01427-9.

## Key summary points

**Aim:**

To examine which signs and symptoms prompt antibiotic prescribing for suspected urinary tract infections in nursing home residents across Europe.

**Findings:**

Signs and symptoms differed markedly between countries. General symptoms were frequently used in some settings, whereas others relied more on urinary tract–specific symptoms or urine changes.

**Message:**

Inconsistent diagnostic practices for suspected urinary tract infections in nursing homes may drive unnecessary antibiotic use and underscore the need for clearer, standardized diagnostic criteria.

**Supplementary Information:**

The online version contains supplementary material available at 10.1007/s41999-026-01427-9.

## Background

Nursing home residents receive a substantial amount of antibiotics [1, 2], significantly more than community-dwelling older adults [3, 4]. The negative consequences of antibiotic use include side effects for the treated residents and the promotion of antimicrobial-resistant bacteria in the resident, the nursing home, and the global community [2, 5]. Antimicrobial resistance is recognized by the World Health Organization as one of the greatest threats to global health [6].

In nursing home residents, urinary tract infections (UTIs) are the most common infections for which antibiotics are prescribed, accounting for nearly half of all antibiotic use in this setting [7]. However, evidence suggests that approximately half of all antibiotic prescriptions for UTIs in nursing home residents are unnecessary, as they do not meet diagnostic criteria, highlighting a significant issue of overdiagnosis [8–11].

To meet the criteria for a UTI diagnosis, two conditions must be fulfilled: the presence of bacteria in the urine (bacteriuria) and the simultaneous presence of relevant symptoms [12]. Bacteriuria is common among nursing home residents, with point prevalences of 25–50% for women and 15–40% for men without indwelling urinary catheters [13]. However, detecting bacteriuria in this population is challenging for two main reasons. First, obtaining a valid urine sample is often difficult because of incontinence, immobility, and cognitive impairment, leading to frequent contamination. Second, urinary dipsticks are commonly used in nursing homes despite not being recommended for this population as they are often positive for reasons other than bacteriuria [12, 14].

Even when urine samples are collected correctly and show significant bacterial growth, many residents have no accompanying symptoms, a harmless condition known as asymptomatic bacteriuria, which should not be treated with antibiotics [8, 12, 13].

Distinguishing between asymptomatic bacteriuria and UTI in nursing home residents can be challenging as there is no established gold standard for which signs and symptoms are required for diagnosis [12–14]. In younger individuals, consensus is that UTI signs and symptoms include dysuria, new-onset frequency, urgency, incontinence, flank pain, suprapubic pain, and gross hematuria. In many older individuals, UTIs may not present with symptoms from the urinary tract but instead manifest as non-specific signs and symptoms, such as altered mental status or fever [12, 15, 16]. Various algorithms have been developed to establish the minimum criteria for initiating antibiotics in nursing home residents with suspected UTI but most are complex and difficult to follow [12, 14, 15, 17–19].

Another challenge in avoiding overdiagnosis of UTI in nursing home residents is that a considerable proportion of residents suffer from dementia or other cognitive impairments. This reduces their awareness and ability to communicate signs and symptoms, making the diagnostic process even more challenging for the staff [14, 20]. In such cases, the diagnosis must be based on a combination of the resident’s reported symptoms and the observations made by the staff and the residents’ relatives.

This study aimed to identify the signs and symptoms leading to antibiotic treatment for UTI in nursing home residents across eight European countries.

## Methods

### Design

This prospective cohort study is part of an EU-funded project entitled “Improving Antibiotic Use in Long-Term Care Facilities by Infection Prevention and Control and Antibiotic Stewardship” (IMAGINE) [21]. The IMAGINE research group consisted of nursing home representatives, microbiologists, geriatricians, and general practitioners. Nursing homes were recruited by convenience sampling from each of the following eight countries: Denmark, Greece, Hungary, Lithuania, Poland, Slovakia, Slovenia, and Spain.

During a three-month period, from 1 st of February to 30th of April 2024, staff at the participating nursing homes filled out a registration chart each time a resident received an oral antibiotic treatment (Additional file 1). The registration was completed by the staff member caring for the resident on the day the antibiotic treatment was initiated (not the prescribing doctor). Ongoing antibiotic prophylaxis and antibiotic courses initiated during hospital admission were not registered.

Since it was the *treatments* and not the residents that were registered, data from residents who received more than one antibiotic treatment during the three-month period were recorded accordingly. Registration was conducted for each *indication* of treatment, meaning that if two types of antibiotics (either two antibiotics from the same group or two different classes of antibiotics) were given for the same indication, only one registration was made. However, if a resident received antibiotics for two different indications at the same time, two separate registrations were made.

Data collection followed the methodology of the Audit Project Odense (APO), a prospective self-registry approach that utilizes a simple reporting template [22].

### Setting

Nursing homes in the participating countries vary considerably in both size and organizational structure. In some countries, all residents have a private room, whereas in others, this is uncommon. Furthermore, staff composition, education, and background—as well as routines and workflow—vary considerably. In some countries, all nursing homes have a designated doctor that prescribes antibiotics for the residents. In other countries, antibiotics are usually prescribed by different doctors outside the nursing homes. In a few countries, courses of certain antibiotics can be initiated by nurses without a doctor’s prescription if predefined criteria are met [7, 23].

### Data

To include signs and symptoms attributed to UTI in nursing home residents in the registration chart, we carried out a narrative review on the diagnosis and overdiagnosis of UTIs in nursing homes [15]. We also sent a questionnaire to all nursing homes included in the study, asking employees associated with the nursing home to list the signs and symptoms they associate with UTI (manuscript awaiting publication). The questionnaire was completed by 286 employees, the vast majority of whom were nurses or other care staff, while eleven were doctors. Further, we interviewed nursing home staff members from each country about various topics regarding the diagnostic process [23].

Based on this information and through repeated meetings, the IMAGINE research group reached consensus on which signs and symptoms to include in the chart. Considerable time was spent discussing the terminology to use, with key considerations being that it should be non-stigmatizing and easily understood by nursing home staff across the participating countries. No medical or technical definitions were applied as staff were not trained in such classifications. Consequently, we did not provide precise definitions—for example, for “Severe cognitive impairment (dementia)”—leaving it to the nursing home staff completing the chart to make this assessment.

The final registration chart consisted of 49 items, covering, among other things, information on the age and sex of the resident, the indication for antibiotic treatment, risk factors for UTI, and both general and urogenital signs and symptoms (Additional file 1).

A pilot test was conducted in November 2023 to ensure that the content of the registration charts was relevant and easy to understand. The registration chart was developed in English and translated into the eight local languages using a standardized forward–backward translation process [24].

### Analysis

Only antibiotic courses given for the indication of UTI were included in the present study. Treatments of residents with an indwelling urinary catheter were excluded from the analyses, as the symptoms and diagnostic criteria differ for this group.

Descriptive statistics was used to analyze differences in signs and symptoms attributed to UTI across countries.

Categorical variables are presented as percentages and metric values as medians. Missing values are reported in the respective tables. The average frequency of each sign and symptom across countries was calculated both weighted and unweighted to account for differences in the number of observations between countries.

Tables and graphical displays were used to assess whether the most significant differences in the frequencies of each sign and symptom between countries were driven by a few outlying nursing homes.

Statistical analysis was conducted using STATA v17.

### Ethics approval and consent to participate

This study adhered to the IMAGINE project protocol, the Declaration of Helsinki, Good Clinical Practice principles, the EU General Data Protection Regulation (2016/679), the Human Research Act as well as other relevant local regulations. Appropriate Regulatory/Ethical approval was sought in each of the countries taking part in the study, and all study procedures started after gaining approval on the basis of the master protocol, translated where necessary to local language.

The study protocol received ethical approval in Spain, the coordinating country, from the Ethics Committee of IDIAP Jordi Gol, Institute of Research in Primary Health Care (ref. 23/080-P) on 12 July 2023.

## Results

### Baseline characteristics

**Fig. 1:**
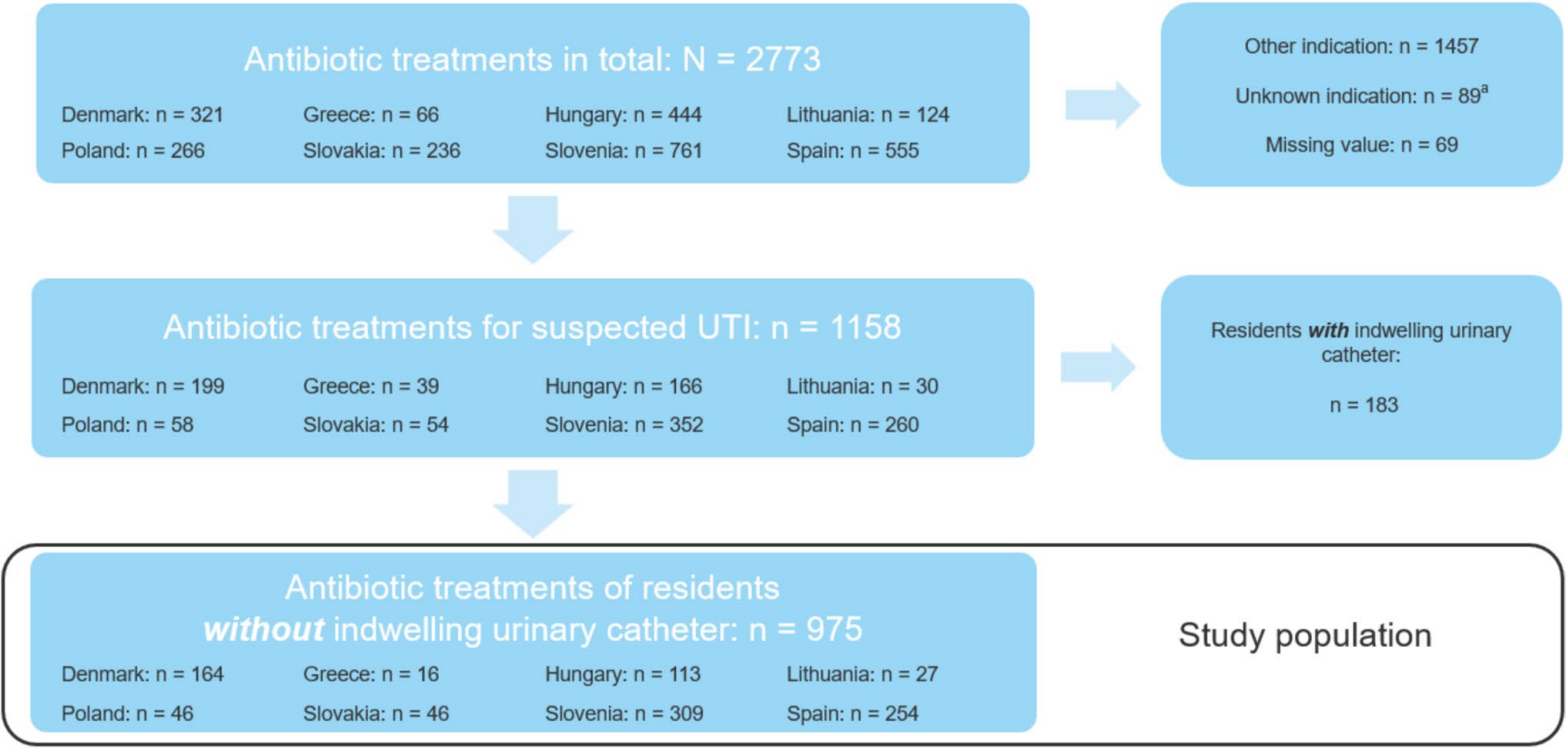
Flowchart illustrating the study population derivation process Abbreviations: UTI, urinary tract infection. **a** “Unknown indication” was selected in the registration chart when the staff member completing the chart was not aware of the reason the doctor had prescribed antibiotics

This study monitored the antibiotic treatments in 109 nursing homes having a total of 9718 residents (Table 1). During the study period, 2773 antibiotic treatments were registered, of which 1158 were administered for suspected UTI. Of these, 975 were given to residents without indwelling urinary catheter, comprising our study population (Fig. 1). The characteristics of the study population and risk factors for developing UTI are presented in Table 1.

**Table 1:** Overview of nursing homes included in the study and the study population’s risk factors for urinary tract infection

	Denmark	Greece	Hungary	Lithuania	Poland^a^	Slovakia	Slovenia	Spain	Total
Number of nursing homes included	15	9	18	15	12	10	15	15	109
Total number of residents	1066	440	1536	779	1441	918	2414	1124	9718
Number of antibiotic treatments for suspected UTI^b ^									
(= study population)	164	16	113	27	46	46	309	254	975
Study population’s risk factors for UTI^c^									
Age (years)^d^, median	86.5	84.5	85	81	67.5	85	86	88	86
Min	64	66	56	54	27	51	56	63	27
Max	102	99	98	97	97	101	105	102	105
Gender (% female)^e^	76	94	88	82	44	85	83	79	76
Severe cognitive impairment (dementia), %	48	44	28	30	9	30	36	55	41
Use of diapers, %	67	75	62	89	44	74	71	85	72
Diabetes, %	10	25	18	26	11	20	15	19	16
History of > = 3 UTIs in the last year, %	29	13	18	7	15	33	21	44	28
Bedridden or use of wheelchair, %	28	38	42	59	37	52	46	38	41

Abbreviations: UTI, Urinary tract infection.

^a^ In Poland, homes for adults with intellectual disabilities were included in the study, explaining the low minimum age and the lower median age compared to the other countries.

^b^ Only among residents without indwelling urinary catheter.

^C^ Likely, some residents were treated more than once.

^d^ Missing values: *n* = 20.

^e^ Missing values: *n* = 10.

### Signs and symptoms attributed to UTI

**Fig. 2:**
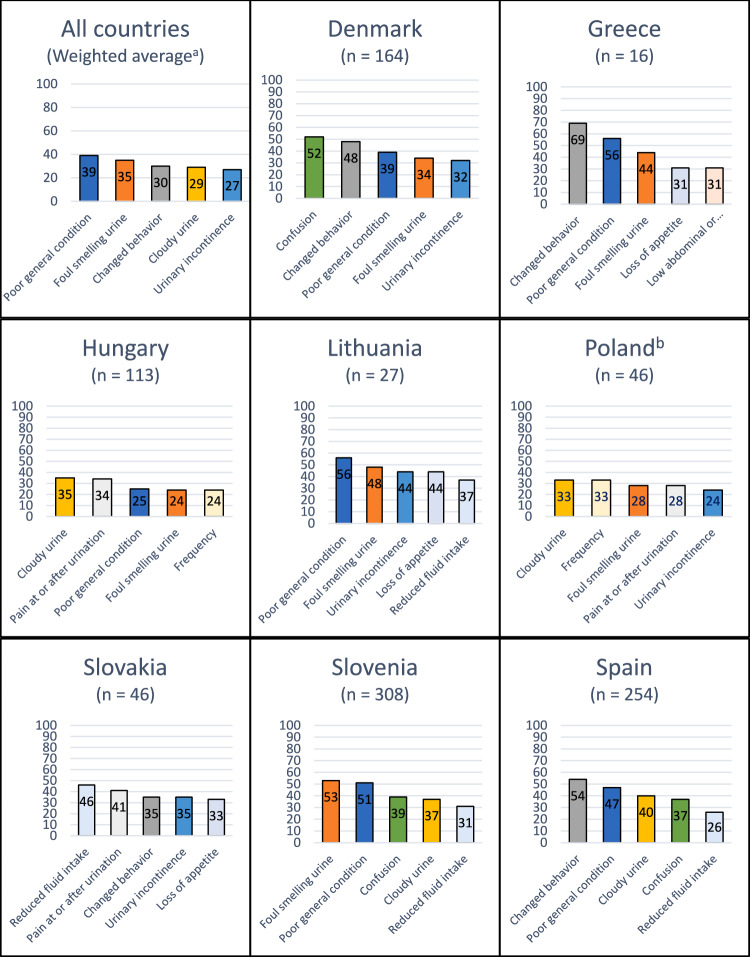
Most frequent registered signs and symptoms in each country, % ^a^ A weighted average is used for the “all countries” graph to give equal importance to each country, accounting for differences in the number of registrations per country. ^b^ In Poland, 24% had urgency and 24% had urinary incontinence, as shown in Table 2. However, only urinary incontinence is displayed here. This is to maintain consistency by showing five bars on each graph, and because urinary incontinence also appears on the “all countries” graph, it is prioritized

Across countries, a considerable variation was observed in the signs and the symptoms attributed to UTI. The percentages of residents recorded with each sign or symptom in each country are presented in Table 2. Overall, for the study population, between 0 and 12 signs or symptoms were checked on the registration chart, with an average of 3.2 signs or symptoms per antibiotic treatment. Figure 2 illustrates the five most common signs or symptoms, overall, and by country.

**Table 2:** Signs and symptoms among residents treated for UTI (new onset or worsening)

	Denmark	Greece	Hungary	Lithuania	Poland	Slovakia	Slovenia	Spain	Total^c^
	*n* = 164	*n* = 16	*n* = 113	*n* = 27	*n* = 46	*n* = 46	*n* = 309	*n* = 254	*n* = 975
General symptoms^a^:									
Fever (temp. ≥ 38 degrees), n (%)	9 (6%)	2 (13%)	7 (6%)	2 (7%)	1 (2%)	2 (4%)	29 (9%)	29 (11%)	81 (8%)
Shaking chills, n (%)	7 (4%)	0 (0%)	1 (1%)	2 (7%)	0 (0%)	4 (9%)	12 (4%)	17 (7%)	43 (4%)
Confusion, n (%)	86 (52%)	4 (25%)	9 (8%)	0 (0%)	3 (7%)	12 (26%)	119 (39%)	95 (37%)	328 (34%)
Poor general condition, n (%)	64 (39%)	9 (56%)	28 (25%)	15 (56%)	10 (22%)	8 (17%)	156 (51%)	119 (47%)	409 (42%)
Changed behavior incl. agitation or apathy, n (%)	79 (48%)	11 (69%)	8 (7%)	2 (7%)	3 (7%)	16 (35%)	50 (16%)	136 (54%)	305 (31%)
Loss of appetite incl. nausea or vomiting, n (%)	19 (12%)	5 (31%)	5 (4%)	12 (44%)	5 (11%)	15 (33%)	48 (16%)	32 (13%)	141 (15%)
Reduced fluid intake, n (%)	27 (17%)	4 (25%)	21 (19%)	10 (37%)	6 (13%)	21 (46%)	96 (31%)	21 (8%)	206 (21%)
None of the above, n (%)	20 (12%)	1 (6%)	55 (49%)	8 (30%)	31 (67%)	15 (33%)	47 (15%)	28 (11%)	205 (21%)
Urogenital symptoms^b^:									
Pain at or after urination, n (%)	35 (21%)	3 (19%)	38 (34%)	9 (33%)	13 (28%)	19 (41%)	55 (18%)	53 (21%)	225 (23%)
Urgency, n (%)	15 (9%)	0 (0%)	24 (21%)	0 (0%)	11 (24%)	14 (30%)	10 (3%)	12 (5%)	86 (9%)
Frequency, n (%)	39 (24%)	0 (0%)	27 (24%)	4 (15%)	15 (33%)	8 (17%)	25 (8%)	37 (15%)	155 (16%)
Urinary incontinence, n (%)	52 (32%)	4 (25%)	26 (23%)	12 (44%)	11 (24%)	16 (35%)	51 (17%)	49 (19%)	221 (23%)
Flank pain, n (%)	15 (9%)	0 (0%)	11 (10%)	2 (7%)	2 (4%)	0 (0%)	24 (8%)	10 (4%)	64 (7%)
Low abdominal or pelvic pain, n (%)	22 (13%)	5 (31%)	16 (14%)	4 (15%)	9 (20%)	8 (17%)	30 (10%)	11 (4%)	105 (11%)
Obvious blood in urine, n (%)	12 (7%)	1 (6%)	2 (2%)	3 (11%)	5 (11%)	0 (0%)	20 (7%)	5 (2%)	48 (5%)
Foul-smelling urine, n (%)	55 (34%)	7 (44%)	27 (24%)	13 (48%)	13 (28%)	12 (26%)	163 (53%)	65 (26%)	355 (36%)
Cloudy urine, n (%)	25 (15%)	4 (25%)	40 (35%)	8 (30%)	15 (33%)	8 (17%)	115 (37%)	101 (40%)	316 (32%)
None of the above, n (%)	24 (15%)	3 (19%)	16 (14%)	4 (15%)	7 (15%)	6 (13%)	39 (13%)	67 (26%)	166 (17%)

Abbreviations: UTI, Urinary tract infection.

^a^ Missing values: 30.

^b^ Missing values: 29.

^c^ Unweighted average.

In some countries, general symptoms dominated, whereas in others, urogenital symptoms were more prevalent. For example, in Spain, four of the five most commonly reported signs and symptoms were general, namely changed behavior, poor general condition, confusion, and reduced fluid intake. None of the five most common signs or symptoms were urogenital in either Spain or Slovenia. In contrast, in Poland, none of the five most common signs or symptoms were general. There, three of the five most commonly reported symptoms were urogenital, namely urinary frequency, pain during or after urination, and urinary incontinence.

Changes in urine characteristics were frequently reported, both overall and within several participating countries. Foul-smelling urine and cloudy urine ranked among the five most commonly reported signs or symptoms overall, as well as in Hungary, Poland, and Slovenia. Slovakia was the only country where neither foul-smelling nor cloudy urine appeared among the top five most commonly reported signs or symptoms.

The most common sign or symptom reported in Denmark was confusion, while in Slovakia it was reduced fluid intake. Neither of these were among the five most common signs and symptoms overall. In the other countries, the most commonly reported sign or symptom (“Changed behavior” in Greece and Spain, “Cloudy urine” in Hungary, “Poor general condition” in Lithuania, “Cloudy urine” and “Frequency” in Poland, and “Foul-smelling urine” in Slovenia) was also among the five most commonly reported overall. Analysis suggested that confusion was a highly prevalent symptom in most nursing homes in Denmark, indicating a generally higher tendency to attribute this symptom to UTI there. In contrast, the high average reporting of reduced fluid intake as a symptom of UTI in Slovakia was largely driven by a very high frequency (88%) in one large nursing home, indicating that this was not a general trend across Slovakia (Additional file II).

Slovakia appeared to be the most divergent from the overall trends as only one of the five most common signs or symptoms reported (changed behavior) was among the top five overall.

## Discussion

Our results underscore the diagnostic complexity and the lack of consensus on which signs or symptoms indicate a UTI in the nursing home population. If clear diagnostic criteria existed and were followed, residents treated with antibiotics for a UTI would exhibit similar signs and symptoms. However, as many as 11 different signs or symptoms rank among the five most common signs or symptoms attributed to UTI across the eight countries. It is striking that among the signs and symptoms typically described in the literature as indicative of UTI—namely those related to the urinary tract—urinary incontinence is the only one that appears among the five most common overall. In all countries except Poland, less than half of the five most frequently reported signs and symptoms are among the literature’s UTI-related signs and symptoms.

Some limitations must be kept in mind when interpreting the results. First, most of the included nursing homes were selected either because the researchers already had an established collaboration with them or because they were geographically close to the research group. Therefore, they were probably not representative of nursing homes in each country, which limits the ability to draw reliable conclusions about the general situation in these countries.

Furthermore, the small number of registrations—especially in some participating countries (e.g., only 16 in Greece)—limits the statistical power. Therefore, we chose a descriptive approach to the data. Larger sample sizes would be necessary to perform analyses with sufficient statistical significance.

Due to the small sample sizes, we did not adjust for differences in the distribution of potential confounders, such as cognitive impairment and diaper use, potentially affecting the recognition of symptoms. For example, differences in the proportion of residents reporting pain are likely related to the prevalence of cognitive impairment, as residents with cognitive impairment may have more difficulty recognizing and communicating pain compared to those without such impairment. Similarly, frequent urination and incontinence are more challenging to observe in residents who use diapers, so a high prevalence of diaper users may be associated with a lower recorded prevalence of frequent urination and incontinence. Conversely, signs, such as blood in the urine, foul-smelling urine, and cloudy urine, may be easier to detect in diaper users than in residents who use the toilet.

Adjusting for these potential confounders requires sufficient data to reliably estimate the relationships between these variables and the outcomes. With a limited number of observations in the individual countries, statistical models become unstable or overfitted when trying to control for multiple confounders, leading to unreliable or misleading results.

By not adjusting for potential confounders, there is a risk that some of the observed variation in signs and symptoms may be due to underlying differences in these characteristics rather than true differences in the outcomes of interest. For example, regarding cognitive impairment, the three countries with the lowest percentages (Poland 9%, Hungary 28%, and Slovakia 30%; see Table 1) are the only ones in which “pain at or after urination” appears among the five most common symptoms (Fig. 2). In terms of diaper use, however, there does not appear to be a systematic connection between its prevalence and the frequency of symptoms. Comparing the country with the lowest prevalence of diaper use (Poland, 44%) with the country with the highest (Lithuania, 89%) reveals that urinary incontinence is recorded much more frequently in Lithuania than in Poland—contrary to what one might expect. On the other hand, cloudy urine is among the top five most common symptoms in Lithuania but not in Poland as expected. Generally, it is unlikely that confounding has compromised the validity of our finding that the attribution of symptoms to UTI varies widely across European nursing homes.

While we collected data on risk factors for individual residents, we did not account for cross-country differences that may have influenced our results. Nursing homes are known to vary widely in structure, organization, and the availability of professional staff, all of which may affect the assessment of residents’ signs and symptoms [23].

It is likely that some antibiotic treatments were not registered, and that some registrations were only partially completed. Staff were asked to register all antibiotic courses over a period of three months, which is a relatively long time and a considerable demand on busy nursing home staff. The research team was aware of this and therefore ensured ongoing dialog with the staff about strategies to ensure all treatments were recorded. For example, this could involve documenting treatments at a specific time of day or assigning a small number of staff members responsible for the registrations. To reduce the number of unregistered treatments and incomplete registrations, nursing homes were reminded several times during the registration period—via phone calls and/or emails from the research team—of the importance of thorough documentation. Unregistered or incomplete treatments are unlikely to have a significant impact on this study, which examines differences in signs and symptoms across countries, as they would probably be evenly distributed and not lead to underreporting of cases with specific signs and symptoms.

The data do not indicate whether the recorded signs and symptoms were first perceived by the residents themselves or by the staff caring for them. By definition, a sign is an objective indicator that can be observed or measured by, for example, nursing home staff, while a symptom is a subjective indicator that only the individual can report [25]. However, in reality, the distinction is often more blurred, also when communicating with the staff [23]. As previously mentioned, depending on the cognitive status of the nursing home residents, the reported signs and symptoms probably often represent a combination of the residents’ own accounts, the staff’s interpretations, and, in some cases, assumptions made by relatives. Some symptoms (e.g., pain) are most likely perceived by the residents and communicated to staff, whereas others (e.g., confusion) are more likely to be observed by staff. Still others (e.g., foul-smelling or cloudy urine) may be equally noticeable to both residents and staff. Therefore, we cannot determine whether observed differences between countries reflect differences in residents’ symptom experiences or differences in the perceptions and practices of the staff. The only thing we can conclude is that the recorded signs and symptoms are considered relevant in relation to UTIs by the staff and doctors in the respective countries; otherwise, the resident would not have been started on antibiotics.

There may be cultural variations in how symptoms are experienced and communicated by nursing home residents across countries, and there may also be cultural differences in the interaction between nursing home residents and staff, which could have influenced our findings [26]. Different perceptions of symptoms across cultures have been described previously, but mostly in relation to psychiatric diagnoses rather than somatic symptoms [27–29]. Cross-cultural differences can be understood through the Health Belief Model, a well-established framework that has been applied across a variety of contexts [30]. According to the model, symptom recognition and reporting are shaped by individuals’ perceptions of susceptibility, severity, benefits of action, and potential barriers. In some countries, for example, residents and staff may have a heightened awareness of UTIs, viewing them to a higher degree as both likely and serious conditions. This can increase the likelihood that general symptoms—such as confusion or changes in behavior—are interpreted as potential signs of UTI and therefore reported. In contrast, in other cultural contexts, such symptoms may be perceived differently and not interpreted as signs of a UTI, and therefore may not lead to antibiotic treatment for that indication.

Furthermore, in an international study like this, some of the variation observed may be due to differences in language. What is considered “confusion” in one language might be described as “changed behavior” in another. This could possibly explain why, for example, confusion is the most frequently reported symptom in Denmark, whereas it is not among the top five symptoms in Greece, which instead has “changed behavior” as the most commonly reported symptom.

Additionally, some signs and symptoms may overlap: if someone is confused, they are likely to also exhibit changed behavior; similarly, if urine is foul-smelling, it is often cloudy as well. Additional analyses show that the most frequent combination of two symptoms in our data was foul-smelling urine and cloudy urine, followed by the combination of confusion and changed behavior. Despite these frequently occurring combinations, there are substantial differences between countries, with some placing greater emphasis on urinary characteristics (foul-smelling and cloudy urine), while others emphasize more general symptoms (e.g., confusion and changed behavior).

Staff were asked to register all new onset or worsening of pre-existing symptoms, and most registrations included more than one sign or symptom. From the data, we cannot determine whether the signs and symptoms were weighted equally in making the diagnosis. For example, some staff members might have recorded both poor general condition and urinary incontinence, but in reality, it may have been the urinary incontinence that prompted suspicion of a UTI, while poor general condition was noted as an incidental finding. Similarly, it is possible that some staff would not suspect a UTI if poor general condition was the only symptom present but would consider it only when combined with one or more other signs or symptoms. This means that signs and symptoms that occur frequently—without necessarily prompting suspicion of UTI—may be overrepresented in our data relative to their true diagnostic importance. We attempted to limit this by emphasizing that only *newly* arisen or worsened signs and symptoms should be recorded.

The challenge of diagnosing UTIs in nursing home residents has been described in several studies, acknowledging the difficulty and lack of consensus [14, 15]. This is primarily due to the high prevalence of asymptomatic bacteriuria compared to other populations, which reduces the value of urine tests, since only a few residents with bacteria in the urine have symptoms and therefore are likely to benefit from antibiotics [12, 14, 15, 17, 18]. Furthermore, nursing home residents’ recognition and communication of symptoms is often influenced by cognitive impairments reducing their validity in diagnosing [12, 20].

What our study adds to the existing literature is a systematic documentation of signs and symptoms attributed to UTIs in nursing home residents, providing an overview of similarities and differences across countries. Our findings confirm the diversity of signs and symptoms and, importantly, show that the classic urogenital symptoms of UTI are less common than more general signs, such as poor general condition and changes in behavior. Furthermore, when nursing home residents have difficulty expressing themselves, staff seem to rely on urine characteristics like foul smell and cloudiness to guide their suspicions.

Our findings reveal that a broad range of symptoms often lead to UTI treatment. In an era where antimicrobial resistance is a major global health threat, the lack of clear diagnostic criteria is concerning. Diagnostic uncertainty frequently results in overdiagnosis, which in turn drives the overuse of antibiotics.

To address antibiotic overuse, future research must focus on strategies to reduce UTI overdiagnosis in nursing home populations. Relying heavily on urine tests is problematic due to the high prevalence of asymptomatic bacteriuria, which can lead to false-positive diagnoses. At the same time, establishing clear guidelines for diagnosing UTIs based on symptoms is challenging in this population.

Future research should prioritize developing the most accurate methods to diagnose UTIs in nursing home residents and strategies to reduce unnecessary antibiotic use. Given that many residents have cognitive impairments and limited ability to communicate symptoms, clear and practical guidelines are essential to support the diagnostic process.

Nursing home staff are central to this effort, as they have daily contact with residents and often initiate the diagnostic process. For example, one study found that an intervention aimed at improving staff knowledge about UTIs and enhancing their communication skills led to a 50% reduction in antibiotic use for UTIs [31]. Therefore, future research should focus on how best to equip and train staff for this vital role. This could include training them to observe and monitor residents suspected of having a UTI, rather than immediately calling a doctor, an approach that could prevent many unnecessary antibiotic treatments [14].

Our study highlights key areas where nursing staff would benefit from additional training, both broadly and within specific countries. Improving communication with residents, as well as the assessment and interpretation of symptoms, are important targets. For example, knowledge that urine characteristics cannot be used as indicators of a UTI, and that general symptoms may have many other causes than a UTI, is essential.

Beyond staff, future research should also examine the roles of nursing home residents themselves and their relatives in the UTI diagnostic process. Currently, little is known about how residents experience and report symptoms or the influence relatives have on diagnosis and treatment decisions.

## Conclusion

A wide range of symptoms leads to the initiation of antibiotic treatment for UTI in nursing homes. They vary considerably across Europe indicating substantial room for improved diagnosing. Expanding the knowledge on how to diagnose UTI in nursing homes, agreeing on guidelines, and training of nursing home staff is crucial to addressing this issue, which is essential for preventing overdiagnosis, antibiotic overuse, and antibiotic resistance.

## Supplementary Information

Below is the link to the electronic supplementary material.

Supplementary file1 (PDF 318 KB)

Supplementary file2 (DOCX 187 KB)

## Data Availability

The dataset used during the current study is available from the corresponding author on reasonable request.

## Abbreviations

UTI: Urinary tract infections
APO: Audit Project Odense
