# Supplementary material for: Signs and symptoms attributed to urinary tract infections in nursing home residents across eight European countries
Source: Eur Geriatr Med. 2026 Feb 13;17(3):1587–97. doi: 10.1007/s41999-026-01427-9 (PMC13309494; doi:10.1007/s41999-026-01427-9)
Supplement: Supplementary file 1 — Supplementary file1 (PDF 318 KB) [file 41999_2026_1427_MOESM1_ESM.pdf]

ID:
9-9-999

IMAGINE - REG. 1:

Treatments with antibiotics in nursing homes (Each line indicates a new treatment)

IMAGINE

	Age (years)	Sex		Indication for treatment (Only 1 X)			Risk factors for urinary tract infection (Min 1 X)							New onset or worsening of preexisting symptoms (Min 1 X)								Urogenital symptoms (Min 1 X)										Diagnostic tests (Min 1 X)						Antibiotics (systemic) (Min 1 X)										Treatment duration
																																Urinary dipstick																
		F	M	1 Urinary tract infection	2 Other indication	3 Unknown indication	4 Indwelling urinary catheter	5 Severe cognitive impairment (dementia)	6 Use of diapers	7 Diabetes	8 History of ≥ 3 UTIs in the last year	9 Bedridden or use of wheelchair	10 None of the above	11 Fever (temp. > 38 degrees)	12 Shaking chills	13 Confusion	14 Poor general condition	15 Changed behavior incl. agitation or apathy	16 Loss of appetite incl. nausea or vomiting	17 Reduced fluid intake	18 None of the above	19 Pain at or after urination	20 Urgency	21 Frequency	22 Urinary incontinence (new or worse)	23 Flank pain	24 Low abdominal or pelvic pain	25 Obvious blood in urine	26 Foul smelling urine	27 Cloudy urine	28 None of the above	29 Nitrite positive	30 Nitrite negative	31 Leucocyte positive	32 Leucocyte negative	33 Urinary dipstick not performed	34 Urine sample sent for culture	35 Pivmecillinam	36 Amoxicillin	37 Amoxicillin + clavulanic acid	38 Fosfomycin	39 Nitrofurantoin	40 Trimethoprim and/or sulfonamides	41 Cephalosporines	42 Quinolones	43 Other antibiotics	44 Do not know	45 Treatment duration - days (999=unknown)
1																																																
2																																																
3																																																
4																																																
5																																																
6																																																
7																																																
8																																																

NH-UK

®Copyright: Audit Projekt Odense, J.B. Winsløws Vej 9A, 1. 5000 Odense C
