# Supplementary material for: Signs and symptoms attributed to urinary tract infections in nursing home residents across eight European countries
Source: Eur Geriatr Med. 2026 Feb 13;17(3):1587–97. doi: 10.1007/s41999-026-01427-9 (PMC13309494; doi:10.1007/s41999-026-01427-9)
Supplement: Supplementary file 2 — Supplementary file2 (DOCX 187 KB) [file 41999_2026_1427_MOESM2_ESM.docx]

Appendix II:

Proportion of residents *at each nursing home*

registered with a certain symptom.

Non-catheterized residents treated for UTI are included.

For each country, each line represents one nursing home.

For each country, the column on the left shows *the number of residents treated for a UTI* at the nursing home. The column on the right shows *the proportion of the UTI treated residents who have been registered with the symptom* (confusion or reduced fluid intake).

For example, in Denmark, the first line indicates that in this nursing home, only one resident was treated for a UTI, and this resident did not have confusion (therefore 0%). The next line indicates that in this nursing home, 8 residents were treated for a UTI, and 25% of these had confusion.

**Symptom: Confusion**

**
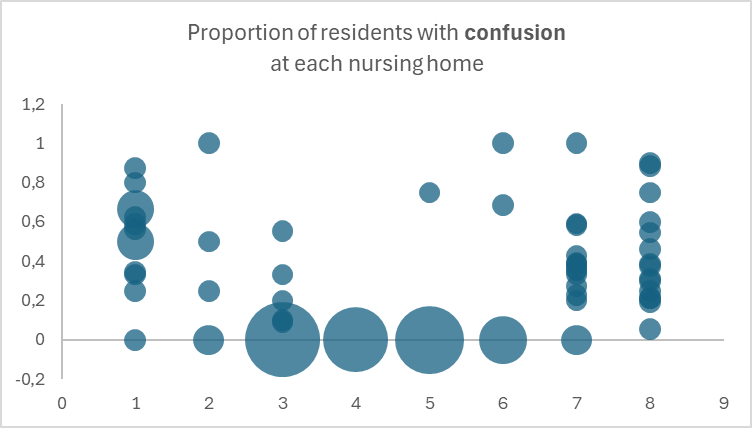
**

Denmark Greece Hungary Lithuania Poland Slovakia Slovenia Spain

**Symptom: Reduced fluid intake**

|  |  |  |
| --- | --- | --- |
|  |  |  |
|  |  |  |
|  |  |  |
|  |  |  |
|  |  |  |
|  |  |  |
|  |  |  |
|  |  |  |
|  |  |  |
|  |  |  |
|  |  |  |
|  |  |  |
|  |  |  |
|  |  |  |
|  |  |  |
|  |  |  |
|  |  |  |
|  |  |  |
|  |  |  |
|  |  |  |
|  |  |  |
|  |  |  |
|  |  |  |
|  |  |  |
|  |  |  |
|  |  |  |
|  |  |  |
|  |  |  |
|  |  |  |
|  |  |  |
|  |  |  |
|  |  |  |
|  |  |  |
|  |  |  |
|  |  |  |
|  |  |  |
|  |  |  |
|  |  |  |
|  |  |  |
|  |  |  |
|  |  |  |
|  |  |  |
|  |  |  |
|  |  |  |
|  |  |  |
|  |  |  |
|  |  |  |
|  |  |  |
|  |  |  |
|  |  |  |
|  |  |  |
|  |  |  |
|  |  |  |
|  |  |  |
|  |  |  |
|  |  |  |
|  |  |  |
|  |  |  |
|  |  |  |
|  |  |  |
|  |  |  |
|  |  |  |
|  |  |  |
|  |  |  |
|  |  |  |
|  |  |  |
|  |  |  |
|  |  |  |
|  |  |  |
|  |  |  |
|  |  |  |
|  |  |  |
|  |  |  |
|  |  |  |
|  |  |  |
|  |  |  |
|  |  |  |
|  |  |  |
